# Sugar Matters: Improving In Vivo Clearance Rate of Highly Glycosylated Recombinant Plasma Proteins for Therapeutic Use

**DOI:** 10.3390/ph14010054

**Published:** 2021-01-11

**Authors:** Sacha Zeerleder, Ruchira Engel, Tao Zhang, Dorina Roem, Gerard van Mierlo, Ineke Wagenaar-Bos, Sija Marieke van Ham, Manfred Wuhrer, Diana Wouters, Ilse Jongerius

**Affiliations:** 1 Department of Immunopathology, Sanquin Research, Landsteiner Laboratory, Amsterdam UMC, University of Amsterdam, 1066 CX Amsterdam, The Netherlands; sacha.zeerleder@insel.ch (S.Z.); R.Engel@sanquin.nl (R.E.); d.roem@sanquin.nl (D.R.); g.vanmierlo@sanquin.nl (G.v.M.); wagenbos@outlook.com (I.W.-B.); m.vanham@sanquin.nl (S.M.v.H.); diana.wouters@rivm.nl (D.W.); 2 Department of Hematology and Central Hematology Laboratory, Inselspital, Bern University Hospital, University of Bern, 3010 Bern, Switzerland; 3 Department for BioMedical Research, University of Bern, 3012 Bern, Switzerland; 4 Center for Proteomics and Metabolomics, Leiden University Medical Center, 2333 ZA Leiden, The Netherlands; T.Zhang@lumc.nl (T.Z.); m.wuhrer@lumc.nl (M.W.); 5 Department of Pediatric Immunology, Reumatology & Infectious Diseases, Emma Children’s Hospital, Amsterdam UMC, 1105 AZ Amsterdam, The Netherlands

**Keywords:** C1-inhibitor, glycosylation, recombinant protein

## Abstract

Correct glycosylation of proteins is essential for production of therapeutic proteins as glycosylation is important for protein solubility, stability, half-life and immunogenicity. The heavily glycosylated plasma protein C1-inhibitor (C1-INH) is used in treatment of hereditary angioedema attacks. In this study, we used C1-INH as a model protein to propose an approach to develop recombinant glycoproteins with the desired glycosylation. We produced fully functional recombinant C1-INH in Chinese hamster ovary (CHO) cells. In vivo we observed a biphasic clearance, indicating different glycosylation forms. *N*-glycan analysis with mass spectrometry indeed demonstrated heterogeneous glycosylation for recombinant C1-INH containing terminal galactose and terminal sialic acid. Using a Ricinus Communis Agglutinin I (RCA_120_) column, we could reduce the relative abundance of terminal galactose and increase the relative abundance of terminal sialic acid. This resulted in a fully active protein with a similar in vivo clearance rate to plasmaderived C1-INH. In summary, we describe the development of a recombinant human glycoprotein using simple screening tools to obtain a product that is similar in function and in vivo clearance rate to its plasma-derived counterpart. The approach used here is of potential use in the development of other therapeutic recombinant human glycoproteins.

## 1. Introduction

The complement system protects us from harm caused by pathogens, immune complexes and apoptopic cells [1]. However, unwanted complement activation is linked to disease development [2]. The complement system is tightly controlled by complement regulators. These natural regulators of the system are considered to be very interesting in therapeutics [3]. One of these complement regulators is C1-inhibitor (C1-INH). C1-INH is a serine protease inhibitor in plasma that plays an important role in the inhibition of proteases of the complement cascade and of proteases of the contact system and the coagulation system [4]. Plasma purified C1-INH was the first FDA-approved protease regulator effectively used in hereditary angioedema (HAE). HAE is a rare disease, caused by partial C1-INH deficiency and characterized by recurrent episodes of severe swelling of, for instance, the face, genitals and gastrointestinal tract. Swelling of the upper airways may occasionally result in asphyxia [5]. One of the treatments for HAE in previous decades was the administration of plasma-derived C1-INH (pdC1-INH); more recently, recombinant C1-INH (rC1-INH, produced in transgenic rabbits) is used [5]. C1-INH is heavily glycosylated, containing at least six *N*-linked and 26 *O*-linked glycosylation sites, and is considered to be one of the most heavily glycosylated proteins in human plasma [6]. Differences in the glycosylation pattern of C1-INH do not seem to influence the protease inhibitory function of C1-INH [7,8]. However, glycosylation does play a major role in clearance. This is shown by the fact that the half-life of rC1-INH produced in transgenic rabbits, containing many mannose residues, is greatly reduced compared to pdC1-INH [9]. The reduction of the half-life of C1-INH is most likely caused by incomplete shielding of glycans with sialic acids and the exposure of N-glycans, which can bind to the asialoglycoprotein and mannose receptors [9,10].

The short half-life of rC1-INH does not have an effect on clinical efficacy when used for treatment of attacks in HAE patients. This indicates that peak plasma levels are more relevant than the half-life of the protein in treatment of this disease [9,10,11]. However, studies in animal models, as well as clinical data, have indicated that treatment with pdC1-INH may also be beneficial in other diseases characterized by complement and contact system activation, such as sepsis, cytokine-induced vascular leakage syndrome, ischemia-reperfusion injuries, acute myocardial infarction, autoimmune hemolytic anemia and renal transplantation [4,12,13,14]. The short half-life of rC1-INH might be an unwanted characteristic for novel clinical applications. The heavily glycosylated complement inhibitory protein factor H, another recombinant complement inhibitory protein in the pipeline for therapeutic purposes, is also under investigation as possibly therapeutic. Similar to rC1-INH, the recombinantly produced factor H also has a potentially disadvantageous short half-life [15,16]. Therefore, strategies to produce recombinant proteins with appropriated glycosylation profiles that resemble the in vivo situation, resulting in a normal clearance rate, are required.

Here, we use C1-INH as a model protein to investigate the molecular basis of the fast clearance rate of recombinant glycoprotein therapeutics in vivo. We show that terminal galactose-containing glycans especially are associated with the fast clearance rate of recombinant proteins. Here, we show that, by using simple purification methods, it is possible to produce recombinant proteins that are fully comparable to their plasma counterparts in both their activity in vitro as well as their half-live in vivo.

## 2. Results

### 2.1. rC1-INH Is Cleared Rapidly in a Biphasic Manner In Vivo

**Figure 1:**
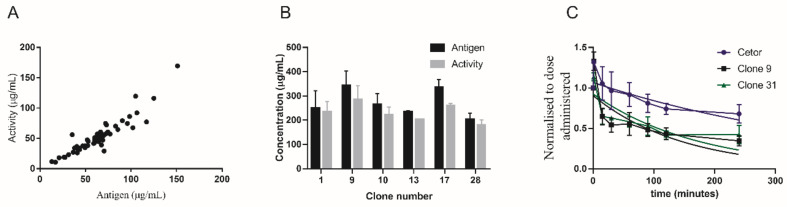
Production levels of recombinant C1-inhibitor (rC1-INH) and half-life in vivo. (**A**) Antigen and activity levels of C1-INH produced by 57 different Chinese hamster ovary (CHO) cell clones. Antigen and activity levels correlate with a correlation efficiency of r = 0.962 *p* < 0.001 (Pearson two-tailed analysis). (**B**) Antigen and activity levels of rC1-INH in culture supernatants of the highest producing C1-INH clones after upscaling. (**C**) In vivo half-life of plasma-derived C1-INH (pdC1-INH) (Cetor) (*n* = 2), rC1-INH isolated from CHO clone 9 (*n* = 6) and rC1-INH isolated from CHO clone 17 (*n* = 2).

It is known that rC1-INH has a very short half-life in vivo due to altered glycosylation patterns causing incomplete shielding of glycans [9,10].To develop a recombinant version of C1-INH with similar function and in vivo clearance kinetics we produced rC1-INH in Chinese hamster ovary (CHO) cells, which are capable of producing similar glycoforms as humans [17]. We expressed rC1-INH with 57 clones. For all clones, the amount of protein and the activity of the produced proteins were determined (Figure 1A and Supplement Figure S1A). This first screening already showed that production of rC1-INH strongly varied per clone. Production levels ranged from 13 µg/mL to as high as 151 µg/mL antigen.

Six of the highest producing clones, namely clone numbers 1, 9, 10, 13, 17 and 28, were chosen for upscaling and further analysis. The upscaling, for all six clones, was efficient with production levels >300 µg/mL for clones 9 and 17 (Figure 1B). Highly pure protein, with a >90% active fraction, was obtained from both purifications (Supplement Figure S1B,C). To estimate the half-life of CHO cell-expressed rC1-INH, the protein pharmacokinetics of clones 9 and 17 were studied in rabbits and compared to the clearance of pdC1-INH (Figure 1C). The rC1-INH preparations cleared faster than the plasma-derived protein, with a half-time of approximately 2 h for the rC1-INH of both clone 9 and clone 17 and 6 h for pdC1-INH (Figure 1C). Surprisingly, an apparently biphasic clearance was observed followed by a slower clearance later on. The biphasic clearance pattern suggests the presence of two differentially clearing protein fractions. In conclusion, we observed that rC1-INH clears much faster from circulation compared to pdC1-INH. However, a biphasic clearance was observed, indicating at least two forms of the recombinant proteins.

### 2.2. Terminal Galactose Screening for rC1-INH

**Figure 2:**
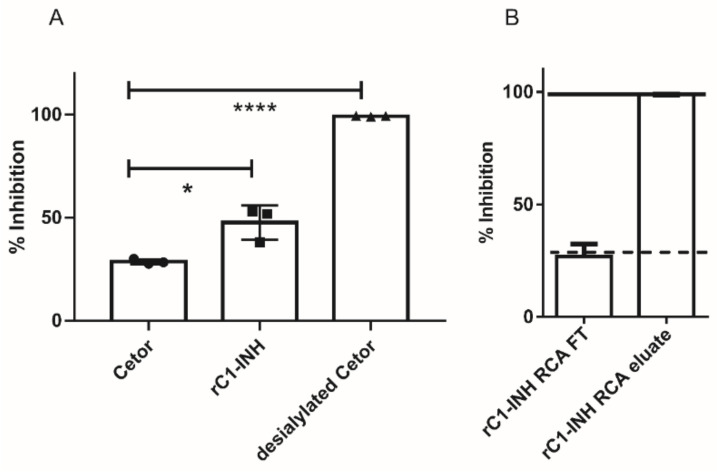
Terminal galactose screening for rC1-INH using RCA_120_. (**A**) Inhibition of desialylated C1-INH to RCA_120_ by pdC1-INH, rC1-INH and desialylated C1-INH as positive control. (**B**) Inhibition of desialylated C1-INH to RCA120 by rC1-INH, passed over an RCA120 column to remove terminal galactoses (rC1-INH RCA FT) and with rC1-INH eluted from the RCA120 column (rC1-INH RCA eluate). We can see that the capacity of inhibition by rC1-INH RCA FT is similar to pdC1-INH (dotted line), while the inhibitory capacity of rC1-INH RCA eluate is similar to desialylated C1-INH (solid line). Values are means ± SD of three independent experiments. * *p* < 0,05; **** *p* < 0,001 (according to two-tailed Students’ T-test).

In vivo, we observed a biphasic clearance of rC1-INH, which indicates at least two forms of the recombinant protein. The presence of glycans expressing terminal galactose can be a cause of rC1-INH clearance from circulation via the hepatic asialoglycoprotein receptors [9,10]. We analyzed the amount of terminal galactose content in an ELISA-based inhibition assay using the lectin Ricinus Communis Agglutinin I (RCA_120_), which binds oligosaccharides with terminal galactose. Figure 2A shows that rC1-INH inhibited binding of RCA_120_ to desialylated C1-INH to a greater extent than pdC1-INH. This indicated that our rC1-INH contained a higher terminal galactose content. Desialylated pdC1-INH served as positive control showing full inhibitory capacity. Next, we depleted rC1-INH with terminal galactose expression proteins via an RCA_120_ coupled column. This terminal galactose-reduced rC1-INH preparation showed a similar inhibition profile as pdC1-INH (shown as dotted line) (Figure 2B). As expected, the rC1-INH present in the eluate of the RCA_120_ column, containing terminal galactoses, can fully inhibit binding of desialylated C1-INH to RCA_120_ similar to desialylated C1-INH itself (solid line).

**Figure 3:**
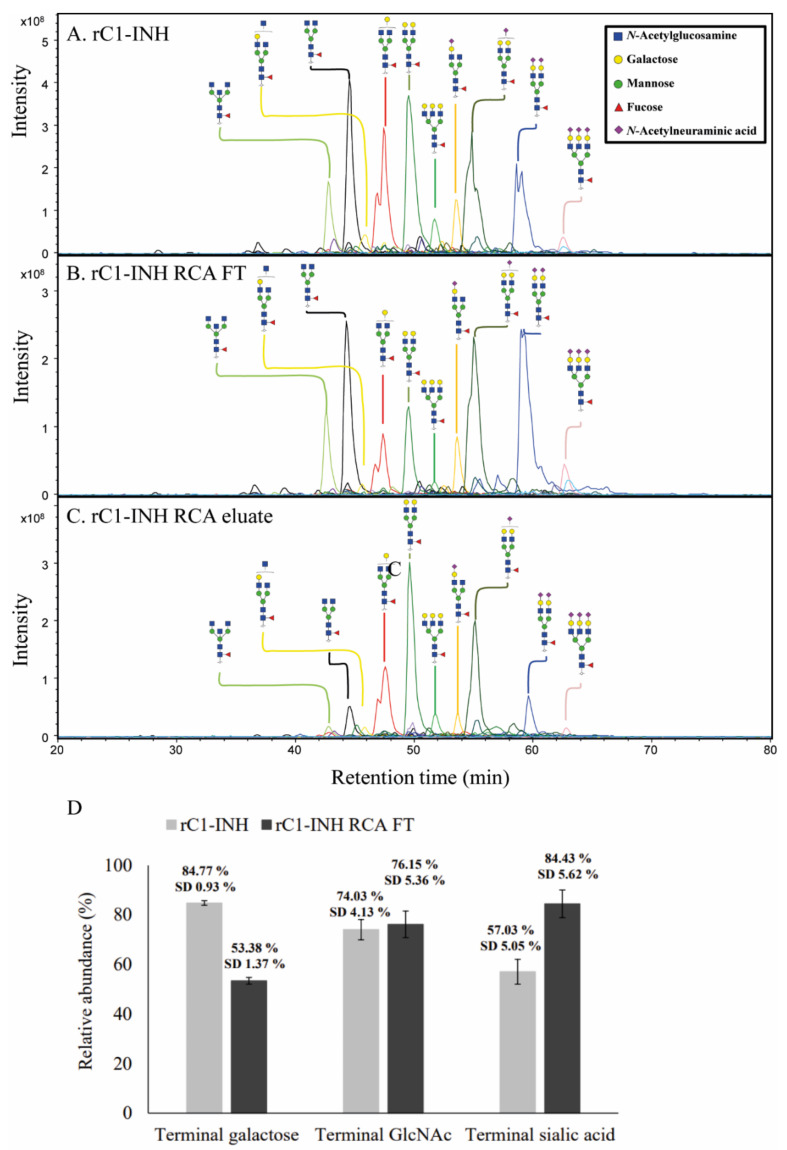
*N*-glycan of rC1-INH, rC1-INH RCA FT and rC1-INH RCA eluate. *N*-glycans were analyzed by PGC nano-LC-ESI-MS/MS and visualized by extracted ion chromatograms (EIC). The *N*-glycosylation profile of rC1-INH RCA FT (**B**) shows a reduced relative abundance of terminal galactose and an increased relative abundance of terminal sialic acid compared to the *N*-glycosylation profile of rC1-INH (**A**). In contrast, rC1-INH eluate displays an increased abundance of terminal galactose and reduced abundance of terminal sialic acid (**C**). The top ten most abundant glycans are annotated per sample and assigned structural schemes. These spectra are representative of three different technical replicates. (**D**) Relative abundance of key structural motifs of rC1-INH and rC1-INH RCA_120_ FT. Relative quantification and structural elucidation of the 40 most abundant glycans was performed per sample (see Supplemental Figure S2). For the relative abundances of the glycan motifs, terminal galactose, terminal GlcNAc and terminal sialic acid, the relative abundance of glycans carrying these motifs were multiplied by the number of these motifs per glycan and summed. Analyses were performed in triplicate and the standard deviation (SD) is given.

Next, as RCA_120_ is known to target N-glycans, we used PGC nano-LC-ESI-MS/MS to analyze the N-glycans on rC1-INH, the rC1-INH RCA FT and the rC1-INH RCA eluate (Figure 3A–C). The rC1-INH glycosylation profile is heterogeneous and includes complex-type di- and triantennary N-glycans with core fucose and various amounts of terminal galactose, (bisecting) GlcNAc and sialic acids (Figure 3A). This is in contrast to pdC1-INH, which has a relatively simple glycosylation profile containing fully sialylated di- and tri- antennary structures and few core fucosylated structures [6]. In agreement with our RCA_120_ results, our PGC nano-LC-ESI-MS/MS analyses of rC1-INH RCA FT showed a reduced relative abundance of N-glycans with terminal galactose and an increased relative abundance of N-glycans with terminal sialic acids compared to nondepleted rC1-INH (Figure 3A,B). Relative quantification and structural elucidation of the 40 most abundant glycans was performed per sample (Supplement Figure S2). α2,3-sialylation was confirmed by MS/MS and sialidase S treatment (Supplement Figure S3).

In summary, we observed a much more heterogeneous glycosylation profile for rC1-INH compared to pdC1-INH, including a higher amount of terminal galactose containing glycans compared to the pdC1-INH. Using an RCA_120_ column, we were able to reduce the amount of terminal galactose content in the rC1-INH fractions.

### 2.3. Removal of Terminal Galactose-Rich Fraction on RCA_120_ Column Does Not Impair C1-INH Function and Improves In Vivo Half-Life of rC1-INH

**Figure 4:**
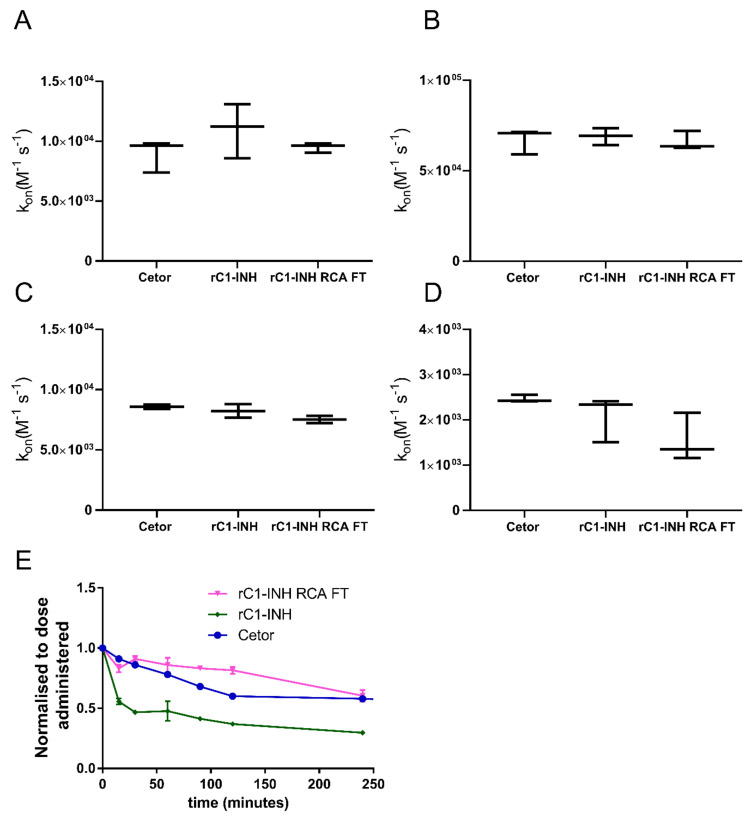
Function and half-life of rC1-INH without terminal galactoses. The association rate constants k_on_ (M^−1^s^−1^) for the inhibition of C1s (**A**), Kallikrein (**B**), Plasmin (**C**) and (**D**) FXIa by pdC1-INH, rC1-INH and rC1-INH RCA FT were determined. Values are means ± SD of three independent experiments, each performed in duplicate. No significant differences were observed. (**E**) The half-lives of pdC1-INH (*n* = 2), rC1-INH (*n* = 3) and rC1-INH RCA FT (*n* = 2) show that the half-lives of pdC1-INH and rC1-INH RCA FT are similar, while the half-life of rC1-INH is clearly reduced.

To ensure that the sialylation-rich fraction of rC1-INH is still active, we measured the kinetics of inhibition of various target proteases by rC1-INH and compared them to those of pdC1-INH. The association rate constants (k_on_) for C1-INH interaction with C1s, FXIIa, kallikrein and plasmin were determined (Figure 4A–D). For all proteases studied, rC1-INH, before and after RCA_120_ depletion, showed k_on_ values that were comparable to pdC1-INH, indicating that the rC1-INH preparations were similar to pdC1-INH in activity. Next, we studied the effect of the removal of galactose-rich fractions on the in vivo clearance rate. Clear improvement in the clearance of the rC1-INH was observed after depletion of the terminal galactose-expressing protein fraction on the RCA_120_ column (Figure 4E). The half-life of pdC1-INH is around 6 h while the half-life of nondepleted rC1-INH is around 2 h (similar to Figure 1C). The half-life of rC1-INH that was passed through an RCA_120_ column was similar to pdC1-INH. In conclusion, we show that the sialylation-rich fraction of rC1-INH is fully functional with a half-life similar to pdC1-INH.

## 3. Discussion

Plasma proteins are interesting candidates for therapeutic purposes, especially in patients with deficiencies or mutations in those proteins. Decades ago, substitution of pC1-INH turned out to be effective in patients suffering from HAE [9,14,18]. HAE is characterized by recurrent episodes of severe swelling, which can result in life-threatening situations [5,19]. C1-INH administration on demand and as prophylaxis reduced the severity and the frequency of attacks, respectively [20]. Other examples of the efficacy of purified plasma proteins in diseases are plasma derived coagulation factors (factor VIII and factor IX) in haemophila [21] or gammaglobulins in primary and secondary humoral immunodeficiencies [22]. Plasma proteins are often highly glycosylated and their half-life significantly decreases in their recombinant substitutes, most likely due to incorrect glycosylation [9,15]. In addition, glycosylation also plays an important role in other biopharmaceuticals and recombinant anti-cancer therapies [23,24]. It is known that plasma proteins reproduced in a recombinant manner, like C1-INH and complement regulator factor H, are cleared much faster from circulation than the respective proteins isolated from plasma [9,15,25]. The half-life of rC1-INH in humans is estimated at approximately 3 h after administration of 100U/kg [9]. The half-life of pdC1-INH is estimated at around 24 h in humans [18,26]. The difference in half-life is explained by the presence of oligomannose and hybrid-type N-linked glycans and a low degree of sialylation resulting in exposure of terminal galactose groups, mannose groups or both [9,27]. Glycosylation of proteins is a result of complex post-translational modification, which can be influenced by the type of host cell and fluctuations in fermentation conditions [28]. Glycosylation patterns of recombinant proteins affect protein solubility, stability, half-life and immunogenicity [28,29,30].

Studies show that a lectin microarray could be used to determine glycan patterns of therapeutic glycoproteins [31]. Here, we showed that removal of the rC1-INH expressing a high level of terminal galactoses resulted in a preparation of rC1-INH with a similar in vivo half-life as pdC1-INH and functional activity towards its target substrates.

Recently, other techniques have been used to optimize glycosylation of recombinant proteins. Glyco-engineered CHO cell lines were used, resulting in fully humanized N-glycosylated profiles of rC1-INH but also of other serum proteins, namely alpha-1-antitrypsin [32,33]. Although this approach resulted in proteins that were similar in glycosylation profile and functions as their plasma-derived counterparts, the half-life in vivo has not been determined. In addition, in an attempt to optimize production of highly glycosylated factor H, the protein was produced in the moss *Physcomitrella*. It is known that plants are able to perform post-translational protein N-glycosylation closely resembling that of humans [25,34]. Although functionally active factor H was produced successfully in high amounts, the half-life of the protein was still reduced compared to plasma-derived factor H [25]. As well as changing the glycosylation profile, other attempts to increase the half-life of proteins have been shown to be very successful. Plasma proteins such as FVIII and FIX have a naturally short plasma half-life (10–14 h and 18–22 h, respectively). Using protein engineering, the proteins were fused to the Fc part of IgG1 to increase the half-life of those proteins. Two of these recombinant coagulation factors, with an extended half-life, are now licensed, namely Fc-fused FIX and FVIII [21,35]. This method will most likely not be optimal for recombinant proteins that are not adequately glycosylated but, in combination with our approach, where we show that we can remove rC1-INH that is not adequately glycosylated, we might end up with a product that has a half-time comparable or even better than its plasma-derived counterpart. Such a product would significantly reduce the treatment burden of patients suffering from HAE.

In summary, we have shown that the presence of terminal galactose groups is indeed responsible for the fast clearance of rC1-INH in rabbits. We presented an elegant and simple method to remove recombinant proteins containing these galactoses from the preparation, which results in rC1-INH with a similar half-life as pdC1-INH. We believe that our described methods can be used to enhance the half-life of other highly glycosylated proteins.

## 4. Materials and Methods

### 4.1. Materials

Ammonium bicarbonate (ABC), Dowex cation-exchange resin (50W-X8), trifluoroacetic acid (TFA), hydrochloric acid (HCl), DL-dithiothreitol (DTT), ammonium acetate, sodium chloride (NaCl) and sodium borohydride (NaBH4) were obtained from Sigma-Aldrich (Steinheim, Germany). The 8 M guanidine hydrochloride (GuHCl) was purchased from Thermo Fisher Scientific. Peptide *N*-glycosidase F (PNGase F) was purchased from Roche Diagnostics (Mannheim, Germany). Glacial acetic acid and potassium hydroxide (KOH) were purchased from Honeywell Fluka. Solid phase extraction (SPE) bulk sorbent Carbograph was obtained from Grace Discovery Sciences (Columbia, MD, USA). HPLC SupraGradient acetonitrile (ACN) was obtained from Biosolve (Valkenswaard, The Netherlands) and other reagents and solvents such as methanol, ethanol and 2-propanol were purchased from Merck (Darmstadt, Germany). MultiScreen^®^ HTS 96-multiwell plates (pore size 0.45 μm) with high protein-binding membranes (hydrophobic Immobilon-P PVDF membrane) and a 96-well PP Microplate were purchased from Millipore (Amsterdam, The Netherlands), conical 96-well Nunc plates from Thermo Scientific (Roskilde, Denmark) and a 96-well PP filter plate from Orochem Technologies (Naperville, IL, USA). Ultrapure water was used for the all preparations and washes, generated from a Q-Gard 2 system (Millipore, Burlington, MA, USA).

### 4.2. Proteins, Antibodies and Substrates

PdC1-INH (Cetor^®^) was obtained from the Sanquin Blood Supply Foundation, The Netherlands. Monoclonal antibody (mAb) RII and polyclonal antibodies against C1-INH were produced previously [36,37]. C1s for kinetic assays and ELISA was purchased from Calbiochem (La Jolla, CA, USA). C1s and pAb against C1-INH were biotinylated using EZ-Link^®^ Sulfo-NHS-Biotin (Thermo Fisher Scientific, Waltham, MA, USA) or Pierce (Rockford, ILL) following the manufacturer protocol. Human Factor XIIa and kallikrein were purchased from Kordia (Leiden, The Netherlands). Agarose-bound RCA_120_ and biotinylated-RCA_120_ were from Vector Labs, USA. Streptavidin-peroxidase was obtained from Amersham Pharmacia Biotech (Uppsala, Sweden). Streptavidin-polymerized peroxidase (poly-HRP) was obtained from Sanquin (Business Unit Reagents, Amsterdam, The Netherlands). Chromogenic substrates S2251, S-2302 and S2366 were from DiaPharma (Beckett Ridge, Ohio, USA); C1s substrate H-D-Val-Ser-Arg.p-NA.2HCl was custom synthesized by Biomatik (Wilmington, DE, USA).

### 4.3. Production and Purification of rC1-INH in CHO Cells

rC1-INH was produced in CHO cell-line CHOExpress^TM^ (ExcellGene, Monthey, Switzerland). Briefly, a CHO-optimized nucleotide sequence was created from the amino acid sequence of the C1-INH (UnitProt P P05155) by DNA2.0 (Menlo Park, CA, USA). After synthesis, the cDNA was sequenced and then subcloned into pXLG6 in DNA2.0 laboratory. Fifty-seven clones of the stable cell-line expressing rC1-INH were grown in small cultures in ProCHO^TM^ medium (ExcellGene). Cell culture supernatants were screened for rC1-INH antigen and active protein levels using ELISA as described previously [8,36,37]. Six of the highest producing clones were chosen for upscaling to 1 L. rC1-INH was purified from the supernatants of two clones (clones 9 and 17) using ion exchange chromatography. Supernatants were diluted in 5 mM trisodium citrate buffer to obtain a final concentration of 5 mM trisodium citrate, pH 5.5. The samples were loaded on a HiPrep 16/10 CM FF column (GE Healthcare, Machelen, Belgium), equilibrated with 5 mM trisodium citrate buffer, pH 5.5. The samples were eluted by raising the pH to 8.5 stepwise with Tris buffer (50 mM, 100 mM NaCl). Peak fractions were collected and the buffer was exchanged to buffer (10 mM, 70 mM NaCl, pH 7.0) with 13 mmol/kg L-alanine, 15 mmol/kg L-valine and 38 mmol/kgL L-threonine trisodium citrate as stabilizers, using a HiPrep 26/10 desalting column (GE Healthcare, Chicago, IL, USA). The samples were aliquoted and stored at −80 °C.

### 4.4. Antigen and Active C1-INH ELISA

The antigen and active levels of rC1-INH were measured using ELISA as described previously [8,38] (see Supplemental Material).

### 4.5. SDS-PAGE

Samples were visualized by gel electrophoresis of SDS-treated samples. Approximately 1 µg of protein per lane diluted in Novex sample buffer was loaded on a Novex 4–12% Bis-Tris gel (Invitrogen, The Netherlands) in MOPS running buffer (Invitrogen, The Netherlands) following the manufacturer protocol. In addition, electrophoresis was performed on NuPAGE^®^ (Invitrogen, Carlsbad, CA, USA) systems according to the instructions of the manufacturer using 4–12% NuPAGE^®^ gels under nonreducing conditions. Gels were developed with a coomassie staining protocol.

### 4.6. Kinetic Analyses of Protease Inhibition

Inhibition of proteases C1s, FXIIa, kallikrein and plasmin by rC1-INH was studied using chromogenic assays under pseudo-first order conditions as described previously [8] (see Supplemental Material).

### 4.7. Animal Experiments

This study was carried out in accordance with the principles of the Basel Declaration and the recommendations of The European Directive 86/609/EEC and the local Animal Welfare Committee of the NKI. The protocol was approved by the local Animal Welfare Committee of the NKI under protocol numbers 2004.009 and 2011.004.

### 4.8. Half-Life of rC1-INH in Rabbits

During the first hour of the study, animals were mildly sedated with either Acepromazine or Domitor, to prevent stress. For CHO-cell produced C1-INH constructs, we used New Zealand White Rabbits weighing <2.5 kg. After sedation, 1 mL of the test sample containing ~1 mg protein was injected intravenously in one ear of the rabbit. Immediately afterwards, 1 mL blood was withdrawn from the other ear, followed by 1 mL withdrawals at 15, 30, 60, 90, 120 and 240 min and then 24 and 48 h. After the last withdrawal, animals were sacrificed by means of a T61 injection. Clotting in blood samples was prevented by use of an anticoagulant mix of 10 mM EDTA (Merck, Darmstadt, Germany), 10 mM benzamidine (Sigma-Aldrich, Steinheim, Germany) and 0.1 mg/mL soybean trypsin inhibitor (Sigma-Aldrich, Steinheim, Germany) (final concentration). After each sample collection, plasma was prepared and stored in liquid nitrogen until further analyses. To compare the clearance rates, antigen levels of the different preparations were normalized to the dose administered, which was calculated by dividing the total antigen administered by the total blood volume of the rabbit. Total blood volume was taken as the weight of the rabbit times 60 mL (=blood volume/kg). Half-life was estimated as the time required for the antigen levels to reach 50% of the administered dose. The half-life was calculated in GraphPadPrism (GraphPad Software Inc., San Diego, CA, USA) with one-phase exponential decay according to the equation t_1/2_ = ln2/k (min).

### 4.9. RCA_120_ Inhibition ELISA 

The amount of terminal galactose content of the various samples was determined by inhibition of the binding of lectin *Ricinus Communis* Agglutinin I (RCA_120_) [39] to desialylated pdC1-INH. Desialylated C1-INH was prepared by treating 180 mg of pdC1-INH in trisodiumcitrate buffer (10mM, pH 6.0) with 0.2 U of neuraminidase coupled to agarose for 6 h at 37 °C, followed by 48 h at 4 °C. The reaction mixture was then centrifuged to remove neuraminidase agarose. Desialylated pdC1-INH was then dialysed against trisodium citrate (10 mM, 70 mM NaCl, pH 7.0 containing 13 mmol/kg l-alanine, 15 mmol/kgL l-valine and 38 mmol/kgL L-threonine). For the inhibition assay, 0.5 µg/mL desialylated pdC1-INH was coated on MaxiSorp^TM^ plates (Thermo Scientific, Roskilde, Denmark) overnight at room temperature. Samples were prepared in a round bottom plate (Greiner, The Netherlands) by incubating equal volumes of 1.25 µg/mL RCA_120_^bt^ and 100 µg/mL of rC1-INH sample for 2 h at room temperature under constant shaking. A calibration curve was similarly prepared by incubating RCA_120_^bt^ with desialylated pdC1-INH (0.2–100 µg/mL). In addition, blanks of only dilution buffer (PBS with 0.1% Tween-20) and only RCA_120_^bt^ in dilution buffer were also taken. At the start of the assay and between steps, MaxiSorp plates were washed five times with wash buffer (PBS containing 0.02% Tween-20). The coated plates were incubated with 100 µL of the sample for 1 h at room temperature under constant shaking followed by incubation with streptavidin-HRP (diluted 1:2000 in dilution buffer) for 1 h at room temperature. Plates were developed using 3,3′,5,5′-tetramethylbenzidine (0.1 mg/mL in 0.11 Na-acetate buffer, pH 5.5, 0.003% H_2_O_2_). The reaction was stopped with 2M H_2_SO_4_ and the absorbance ratio of 450 to 540 nm was determined. The percentage inhibition of each sample was calculated by normalizing the absorbance ratio 450/540 nm for each sample between the absorbance obtained for 100% inhibition (absorbance ratio of buffer blank) and 0% inhibition (absorbance ratio of RCA_120_^bt^ sample only).

### 4.10. Separation of Terminal Galactose-Rich Fraction on RCA_120_ Column

Agarose-bound RCA_120_ lectin was used to separate CHO-produced rC1-INH molecules with terminal galactose from the rC1-INH clone 17. A 4 mL column of RCA_120_-agarose was prepared. The column was equilibrated with 60 mL of 10 mM sodiumcitrate buffer (pH 7.0, with 70 mM NaCl, 13 mmol/kg L-alanine, 15 mmol/kgL L-valine and 38 mmol/kgL L-threonine as stabilizers). A total of 5 ml of a sample containing 2–6 mg protein was applied to the column. The flow-through from the sample was reapplied twice to the column to ensure maximal binding of the terminal galactose-containing protein population to the lectin. The protein fraction that flowed through after the third application was collected. The column was then washed with 5 mL equilibration buffer and flow-through was collected separately. After washing the column with 20 mL PBS, bound rC1-INH was eluted with 200 mM galactose in four 1.5 mL fractions. The flow through, wash and eluted fractions were analyzed for total antigen and activity levels and for their sialic acid content.

### 4.11. Preparation of N-Glycan Alditols Released from rC1-INH and RCA_120_ Depleted rC1-INH

*N*-glycans were released from rC1-INH and RCA_120_-depleted rC1-INH and reduced as previously described [40] (see Supplemental Material).

### 4.12. Analysis of N-Glycan Alditols Released from rC1-INH and RCA_120_-Depleted rC1-INH Using PGC Nano-LC-ESI-MS/MS

The analysis of *N*-alditols was performed following a method described previously [40]. Measurements were performed on an Ultimate 3000 UHPLC system (Dionex/Thermo) equipped with a home-packed PGC trap column (5 μm Hypercarb, 320 μm × 30 mm) and a home-packed PGC nano-column (3 μm Hypercarb 100 μm × 150 mm) coupled to an amaZon ETD speed ion trap (Bruker, Bremen, Germany). Mobile phase A consisted of 10 mM ABC, while mobile phase B was 60% (*v*/*v*) acetonitrile/10 mM ABC. To analyze glycans, 2 μL of each 10 μL sample derived from 20 µg rC1-INH protein was injected and trapped on the trap column using a 6 μL/min flow of 2% B for 5 min. Separation was achieved with a multistep gradient: 2–9% B in 1 min and 9–49% B in 80 min followed by a 10 min wash step at 95% B at a flow of rate of 0.6 μL/min. The column was held at a constant temperature of 45 °C. Ionization was achieved using the nanoBooster source (Bruker, Bremen, Germany) with a capillary voltage of 1000 V applied and a dry gas temperature of 280 °C at 5 L/min and isopropanol-enriched nitrogen at 3 psi. MS spectra were acquired within an m/z range of 500–1850 in enhanced mode using negative ion mode; smart parameter setting (SPS) was set to *m*/*z* 1200. MS/MS spectra were recorded using the top three highest intensity peaks.

Structures of detected glycans were studied by MS/MS in negative mode as well as through additional α2-3 neuraminidase treatment [41]. Glycan structures were assigned on the basis of the known MS/MS fragmentation patterns in negative ion mode [42,43,44], elution order and general glycobiological knowledge, with the help of Glycoworkbench [45] and Glycomod [46] software. Relative quantification of individual glycans was performed by normalizing the total peak area of all glycans within one sample to 100%. Relative abundances of specific glycan structures were grouped by summing the relative abundances of each glycan multiplied by the number of motifs per glycan.

## Data Availability

The data presented in this study are available in this article and associated supplemental material.

## Supplementary Materials

The following are available online at https://www.mdpi.com/1424-8247/14/1/54/s1, experimental descriptions of antigen and active C1-INH ELISA, kinetic analyses of protease inhibition, and preparation of *N*-glycan alditols released from rC1-INH and RCA_120_ depleted rC1-INH. Figure S1: Expression levels and purification of rC1-INH by CHO-cells, Figure S2: Relative quantification of top 40 most abundant *N*-glycans derived from rC1-INH, rC1-INH RCA FT and rC1-INH RCA eluate on PGC nano-LC-ESI-MS/MS, Figure S3: Analysis of *N*-glycans with sialic acids derived from rC1-INH with and without sialidase S treatment.
