# Supplementary material for: Sugar Matters: Improving In Vivo Clearance Rate of Highly Glycosylated Recombinant Plasma Proteins for Therapeutic Use
Source: Pharmaceuticals (Basel). 2021 Jan 11;14(1):54. doi: 10.3390/ph14010054 (PMC7826800; doi:10.3390/ph14010054)
Supplement: Supplementary file 1 [file pharmaceuticals-14-00054-s001.pdf]

Supplemental Material

Antigen and active C1-INH ELISA. The antigen and active levels of rC1-INH were measured using ELISA as described before [1,2]. In short, C1-INH was bound to mAb RII against human C1-INH, coated on Maxisorp™ plates (Nunc, USA), and detected with biotinylated C1s. Notably, this assay is an end-stage assay, that measures the number of functional C1-INH molecules rather than the kinetics of the interaction between C1s and C1-INH. C1-INH multimers were detected in an ELISA with mAb RII both as coating and as detecting antibody. Maximally multimerized plasma derived C1-INH was used as standard and set to 100 arbitrary units (AU). PBS/0.1% Tween was used as the buffer for all incubation steps in the ELISAs.

Kinetic analyses of protease inhibition: Inhibition of proteases C1s, FXIIa, kallikrein and plasmin by rC1-INH was studied using chromogenic assays under pseudo-first order conditions as described earlier [1]. In brief, a fixed concentration of protease was added to varying rC1-INH concentrations in the presence of relevant chromogenic substrate in 96 wells microtiter plates and absorbance at 405 nm was measured for up to 5 hours using Thermo Scientific Multiskan GO plate reader, maintained at 37^0 C. All wells were layered with 25 μl mineral oil to prevent evaporation. Phosphate buffer saline (pH 7.4) with 0.1% Tween-20 was used as the assay buffer. All measurements were performed in duplicate and repeated thrice. rC1-INH sample concentrations were ascertained using absorbance at 280 nm. Progress curves obtained for the various conditions were fitted to the integrated rate equation for slow binding inhibition $A = v_s t + (v_0 - v_s)(1 - e^{-k_{obs} t})/k_{obs} + A_0$, where A is absorbance at 405 nm, v_0 is the initial rate, v_s is the final steady state rate, *t* is time, and k_{obs} is the apparent rate constant, by nonlinear regression analysis. (add ref). Plots of k_{obs} values against inhibitor concentration [I] were used to obtain the association rate constant (k_{on}) using the relationship $k_{obs} = k_{off} + k_{on} [I]/(1+[S]/K_m)$, where k_{off} is the dissociation rate constant, *[S]* is the concentration of substrate used and K_m is the Michaelis-Menten constant for a given protease – substrate pair. *Km* values were obtained separately for each substrate – protease pair under the present experimental conditions. All data was analyzed using GraphPad Prism (GraphPad Software, Inc. USA).

Preparation of *N*-glycan alditols released from rC1-INH and RCA_{120} depleted rC1-INH. *N*-glycan were released from rC1-INH and RCA_{120} depleted rC1-INH and reduced as previously described [3]. In brief, 20 μg of rC1-INH protein were applied to the hydrophobic Immobilon-P PVDF membrane in a 96-well plate format. Protein denaturation was achieved by applying 75 μL denaturation mix (72.5 μL 8 M GuHCl and 2.5 μL 200 mM DTT) in each well, followed by shaking for 15 min and incubating at 60°C in a moisture box for 30 min. Subsequently the unbound material was removed. *N*-glycans were released by adding PNGase F (2 U of enzyme diluted with water to 15 μL) to each well and incubating overnight at 37˚C. Released *N*-glycans were collected from the PVDF plate by centrifugation, and the glycosylamine forms of the released *N*-glycans were hydrolyzed by adding 20 μL of 100 mM ammonium acetate (pH 5) and incubating at RT for 1 h, followed by drying in a vacuum centrifuge (Eppendorf, Hamburg, Germany) at 35˚C. Collected *N*-glycans were then reduced and desalted followed by a PGC clean-up [3,4]. Samples were dried by vacuum centrifugation in polymerase chain reaction (PCR) plates and dissolved in 10 μL of water for PGC nano-LC-ESI-MS/MS analysis.

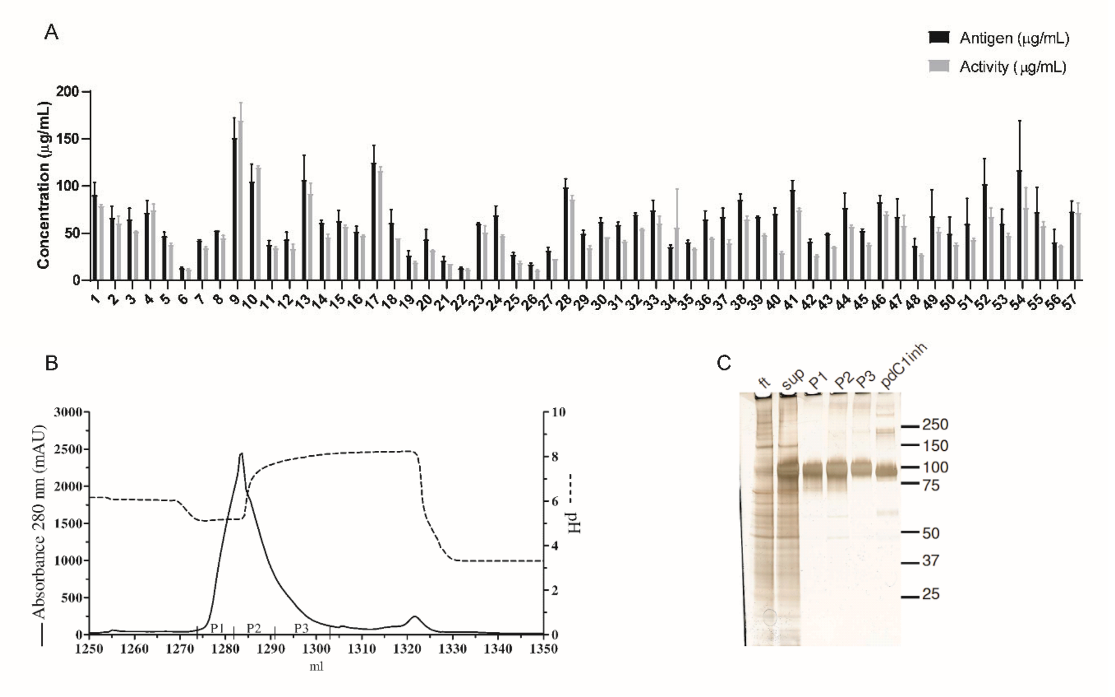

Sup Figure. 1: Expression levels and purification of rC1-INH by CHO-cells. (**a**) Expression levels of rC1-INH antigen and activity of each of the 57 different CHO-cell clones. (**b**) Elution profile of rC1-INH produced by clone 17 on a CM FF Sepahrose column. Solid line: absorbance at 280nm; dashed line: pH changes; P1, P2, P3: peak fractions collected separately. (**c**) SDS-PAGE analysis of protein purity at different steps of purification. For comparison pdC1-INH profile is also shown. 'ft' = flow through; 'sup' = supernatant (the start material), P1, P2 and P3 = the three different fractions collected in (B) after buffer exchange to trisodium citrate buffer.

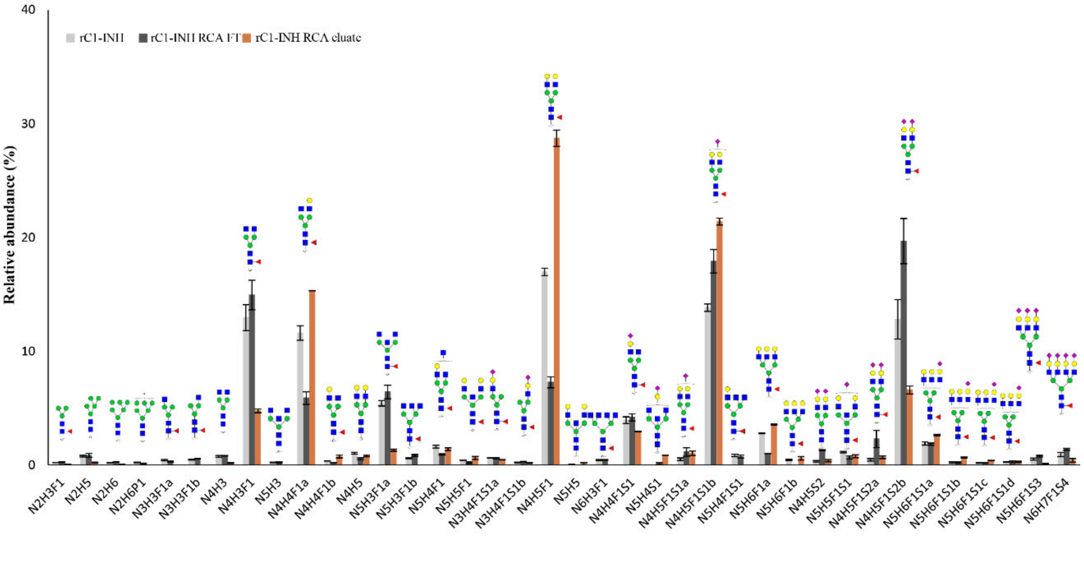

Sup Figure. 2: Relative quantification of top 40 most abundant *N*-glycans derived from rC1-INH, rC1-INH RCA FT and rC1-INH RCA eluate on PGC nano-LC-ESI-MS/MS (Relates to Fig. 3a**).** Relative abundance of individual *N*-glycan derived from 20 µg rC1-INH in three samples on PGC nano-LC-ESI-

MS/MS in negative ion mode (displayed as mean relative abundance plus standard deviation; N=3). Blue square: *N*-acetylglucosamine, yellow circle: galactose, green circle: mannose, red triangle: fucose, pink diamond: *N*-acetylneuraminic acid, H: hexose, N: *N*-acetylhexosamines, F: fucose, S: *N*-acetylneuraminic acid. a, b, c, d: different isomer format.

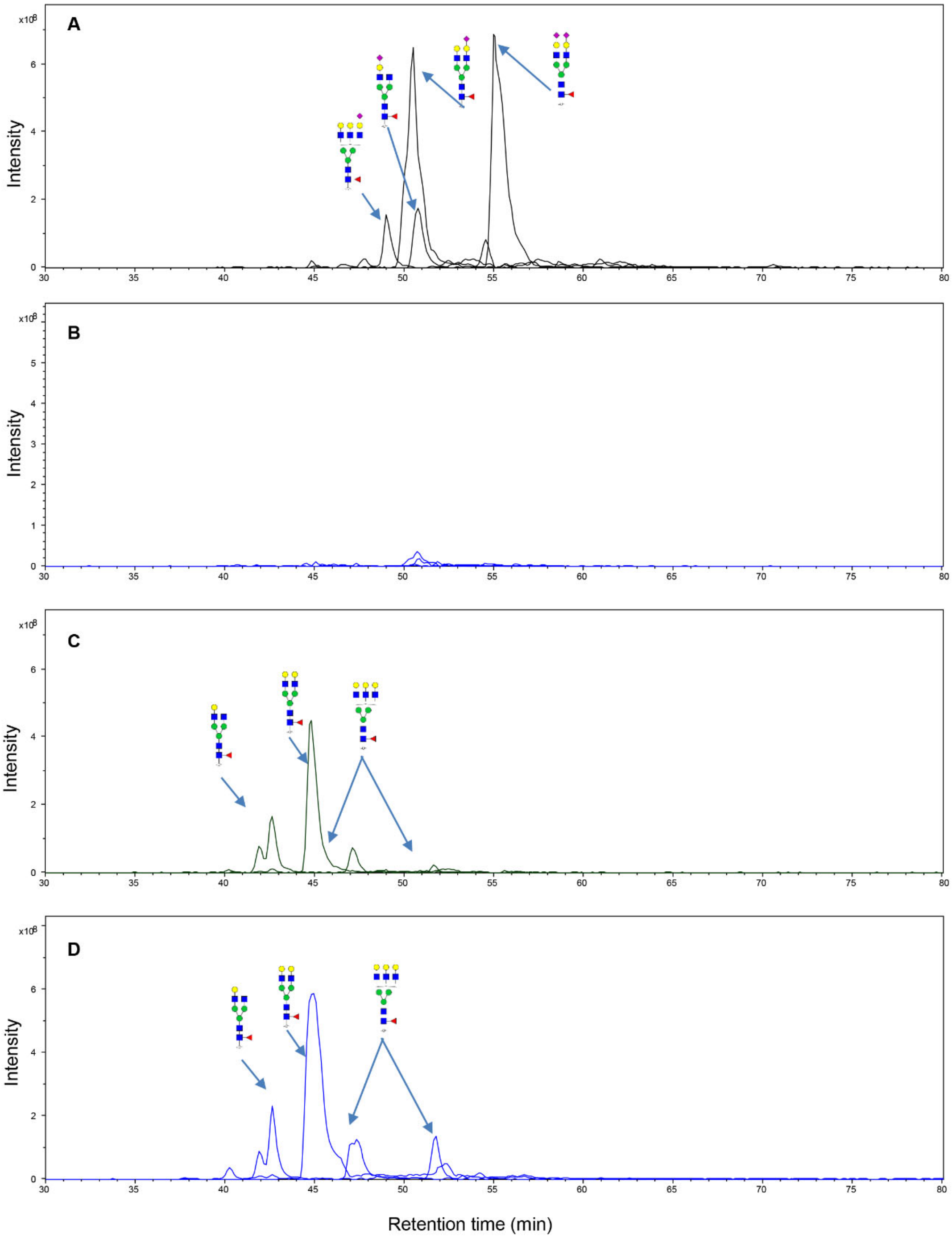

Sup Figure. 3: Analysis of *N*-glycans with sialic acids derived from rC1-INH with and without sialidase S treatment. (**a**) Combined EICs of *N*-glycans with sialic acid derived from rC1-INH before sialidase S treatment. (**b**) Combined EICs of *N*-glycans with sialic acid derived from rC1-INH after sialidase S

treatment. (**c**) Combined EICs of *N*-glycans without sialic acid (potential products after sialidase S treatment) derived from rC1-INH before sialidase S treatment. (**d**) Combined EICs of *N*-glycans without sialic acid (potential products after sialidase S treatment) derived from rC1-INH after sialidase S treatment. Blue square: *N*-acetylglucosamine, yellow circle: galactose, green circle: mannose, red triangle: fucose, pink diamond: *N*-acetylneuraminic acid.

1. Bos, I.G.; de Bruin, E.C.; Karuntu, Y.A.; Modderman, P.W.; Eldering, E.; Hack, C.E. Recombinant human c1-inhibitor produced in pichia pastoris has the same inhibitory capacity as plasma c1-inhibitor. *Biochim Biophys Acta* **2003**, *1648*, 75-83.
2. Bos, I.G.; van Mierlo, G.J.; Bleeker, W.K.; Rigter, G.M.; te Velthuis, H.; Dickneite, G.; Hack, C.E. The potentiation of human c1-inhibitor by dextran sulphate is transient in vivo: Studies in a rat model. *Int Immunopharmacol* **2001**, *1*, 1583-1595.
3. Zhang, T.; Madunic, K.; Holst, S.; Zhang, J.; Jin, C.; Ten Dijke, P.; Karlsson, N.G.; Stavenhagen, K.; Wuhrer, M. Development of a 96-well plate sample preparation method for integrated n- and o-glycomics using porous graphitized carbon liquid chromatography-mass spectrometry. *Mol Omics* **2020**.
4. Jensen, P.H.; Karlsson, N.G.; Kolarich, D.; Packer, N.H. Structural analysis of n- and o-glycans released from glycoproteins. *Nat Protoc* **2012**, *7*, 1299-1310.
